# Decoding flavor evolution during refining of DHA-rich *Schizochytrium* oil: a multidimensional framework integrating sensory evaluation and volatile metabolomics

**DOI:** 10.1016/j.fochx.2026.104255

**Published:** 2026-07-28

**Authors:** Mingjie Li, Guanzhong Xiang, Hanle Yuan, Yuqi Zhang, Yunpeng Ding, Xiaolong Yang, Pan Gao, Jiaojiao Yin, Xinghe Zhang, Zhuolong Guan, Lei Tan, Hanhua Lou, DongPing He, Wu Zhong, Yacheng Hao

**Affiliations:** a Key Laboratory of Edible Oil Quality and Safety, State Administration for Market Regulation, Key Laboratory for Deep Processing of Major Grain and Oil of Ministry of Education in China, College of Food Science and Engineering, Wuhan Polytechnic University, Wuhan 430023, China; b Wuhan Institute for Food and Cosmetic Control, Wuhan 430012, China; c Hubei Fuxing Biotechnology, Hanchuan 431608, China; d Hubei Bashan Food Co., Ltd., Zhushan County, Hubei 442200, China

**Keywords:** *Schizochytrium* Oil, Flavor, Volatile substances, Refining, Deodorization

## Abstract

The influence of refining conditions on flavor evolution of *Schizochytrium* oil remained unclear. Crude oil exhibited strong fishy and sour odors, with 3-octen-2-one and 5-ethyl-2(5H)-furanone as key off-flavor contributors. Degumming and deacidification marginally improved these odors, while decolorization paradoxically intensified fishiness by selective removal of masking compounds. Deodorization was critical: 170 °C had limited effects; optimal performance at 190 °C/30 min largely eliminated off-flavors and allowed a mild pleasant aroma to emerge. Under this condition, eight volatile metabolites were significantly reduced, serving as a practical biomarker panel. Notably, non-key odorants with rOAV < 1 also contributed to flavor via matrix effects. In contrast, 210 °C induced thermal degradation, causing a rebound of aldehydes and ketones that compromised flavor quality. This reveals a biphasic mechanism: volatile removal below 190 °C versus off-flavor rebound above 210 °C, offering both mechanistic insight and practical utility for oil refining.

## Highlights

• Biphasic thermal transition governs flavor evolution during oil deodorization: volatiles removed <190°C, off-flavors rebound >210°C.
• Deodorization at 190°C/30 min removes suppressive off-odorants, allowing latent pleasant aromas to become detectable.
• A biomarker panel of eight volatile metabolites is proposed for monitoring deodorization endpoint in oil refining.
• Untargeted metabolomics with sensory flavoromics links metabolites to attributes and reveals off-flavor contributions from non-key odorants (rOAV<1).

## 1 Introduction

Docosahexaenoic acid (DHA), a long-chain omega-3 polyunsaturated fatty acid, plays a critical role in infant neurodevelopment and adult cardiovascular health (Salmazo et al., 2023). Conventional fish oil sources are constrained by sustainability bottlenecks and risks of contaminant accumulation (Nguyen et al., 2018)**.** This prompted *Schizochytrium* oil to emerge rapidly as a high-purity and scalable alternative lipid (Li et al., 2023b). The DHA content of *Schizochytrium* oil often exceeds 40% of total fatty acids, with a fatty acid profile highly concentrated in C16:0, DPA, and DHA (Afnovandra et al., 2021). *Schizochytrium* oil has been widely applied in infant formula, functional foods, and clinical nutrition (Ryckebosch et al., 2014). However, the inherent sensory defects of crude *Schizochytrium* oil, severely restrict its high-value applications (Nm & Marie, 2021).

These undesirable flavors originate from multiple classes of volatile organic compounds generated jointly through lipoxygenase pathways, lipid oxidation, and Maillard reactions (Li et al., 2023a). These compounds include short-chain aldehydes, ketones, and heterocyclic compounds with extremely low odor thresholds (Yuan et al., 2022). Traditional flavor characterization methods either rely on sensory descriptions that cannot elucidate molecular mechanisms (Hrncirik et al., 2011). Methods limited to preset targets also struggle to capture the complete landscape and dynamic evolution of off-flavor formation (Drover et al., 2010). Volatile metabolomics, through untargeted full-spectrum scanning and high-throughput analysis (Jim et al., 2013), deconstructs flavor into a dynamic chemical network composed of hundreds of molecules (Jensen et al., 2010). This approach can fully capture the multidimensional off-flavors driven by oxidative, enzymatic, and thermal processes (Tahir & Siddiq, 2025). Integrating this technique with sensory information via chemometric correlation, namely the flavoromics strategy, establishes direct causal links between molecular abundance and olfactory perception (Fleith & Clandinin, 2005). It precisely anchors key off-flavor active substances, excludes chemical noise, and provides clear molecular targets for flavor regulation (Nm & Marie, 2021).

Current research on refining effects has mostly focused on nutritional and physicochemical parameters (Chen et al., 2026)**.** The continuous transformation patterns of volatile compounds throughout the entire refining chain, as well as the key odor-active substances driving characteristic off-flavors, remain to be systematically elucidated (Chang et al., 2020) Therefore, this study aims to systematically decode the flavor chemical evolution throughout the entire refining process of *Schizochytrium* oil. The core innovation lies in the first-time deep coupling of untargeted volatile metabolomics with sensory-oriented analysis, constructing a dynamic flavoromics framework for the complete refining chain of *Schizochytrium* oil. This framework synchronously tracks the fluctuation of chemical signals and the reshaping of sensory quality across successive refining stages, breaking through traditional discrete research paradigms and establishing a causal bridge between molecular transformation and flavor improvement. This integrated strategy not only precisely identifies key off-flavor substances and their evolution patterns, but also provides a theoretical foundation for precise flavor regulation of algal oil. It holds significant scientific importance for promoting the directed manufacturing and industrial upgrading of high-quality DHA algal oil.

## 2 Materials and methods

### 2.1 Materials

The crude algal oil from *Schizochytrium* sp. was provided by Hubei Fuxing Biotechnology Co., Ltd. (China). Analytical grade reagents (methanol, glacial acetic acid, ethyl acetate, potassium iodide, soluble starch, sodium thiosulfate, potassium hydroxide, and phosphoric acid) and chromatographic grade solvents (n-hexane, isopropanol, and acetone) were purchased from Sinopharm Chemical Reagent Co., Ltd. (China). Fatty acid standards and benzo[a]pyrene were obtained from Supelco Chemical Co. Ltd. (Shanghai, China).

### 2.2 Refining procedure

#### 2.2.1 Degumming

A 200 mL aliquot of crude oil was placed in a conical flask and heated in a water bath at 75 °C with stirring at 120 r/min. Then, 5% (*w*/w) of distilled water (75 °C) and 0.1% (*v*/v) of phosphoric acid were added. The water bath temperature was subsequently raised to 90 °C. When obvious phospholipid aggregates appeared, the stirring speed was reduced to 30 r/min. After the phospholipid aggregated and the oil phases were clearly separated, the stirring was stopped. The mixture was allowed to stand for 4 h, or centrifuged at 4000 r/min to collect the upper oil layer as degummed oil.

The degumming rate P (%) was calculated using Eq. (2-1):

(2-1) $$P\left(\%\right)=\left[\left(M_{1}-M_{2}\right)\times 100\right]/M_{1}$$

where M_1_ was the weight of oil before degumming (g), and M_2_ was the weight of oil after degumming (g).

#### 2.2.2 Deacidification

The degummed oil was heated to 40 °C with stirring at 200 r/min, and 7°Bé NaOH solution was added dropwise. After the complete addition of alkali, the stirring speed was reduced to 30 r/min, and the mixture was maintained at 70 °C for 30 min. The mixture was centrifuged at 5000 r/min to remove the soapstock. The oil phase was washed with ultrapure water repeatedly until the waste water gave no color change with phenolphthalein. The resulting oil was designated as deacidified oil.

The alkali dosage was calculated using Eq. (2-2):

(2-2) Alkali dosage=Theoretical alkali+Excess alkali

$$\text{Theoretical alkali}=\left(7.14\times m_{1}\times \mathrm{AV}\right)/\left(1000\times C\right)$$

$$\text{Excess alkali}=m_{1}\times W$$

where m_1_ was the oil weight (g), AV was the acid value of the oil (mg/g), W was the mass fraction of excess alkali relative to the oil, and C was the mass fraction of NaOH.

#### 2.2.3 Decolorization

The deacidified oil was heated to 70 °C with stirring at 120 r/min, after which 3% (*w*/w) bleaching clay relative to the oil weight was added. The temperature was then raised to 90 °C and maintained for 1 h with stirring at 200 r/min. After cooling to room temperature, the mixture was centrifuged at 5000 r/min, and the upper oil layer was collected as decolorized oil.

#### 2.2.4 Deodorization

**Fig. 1:**
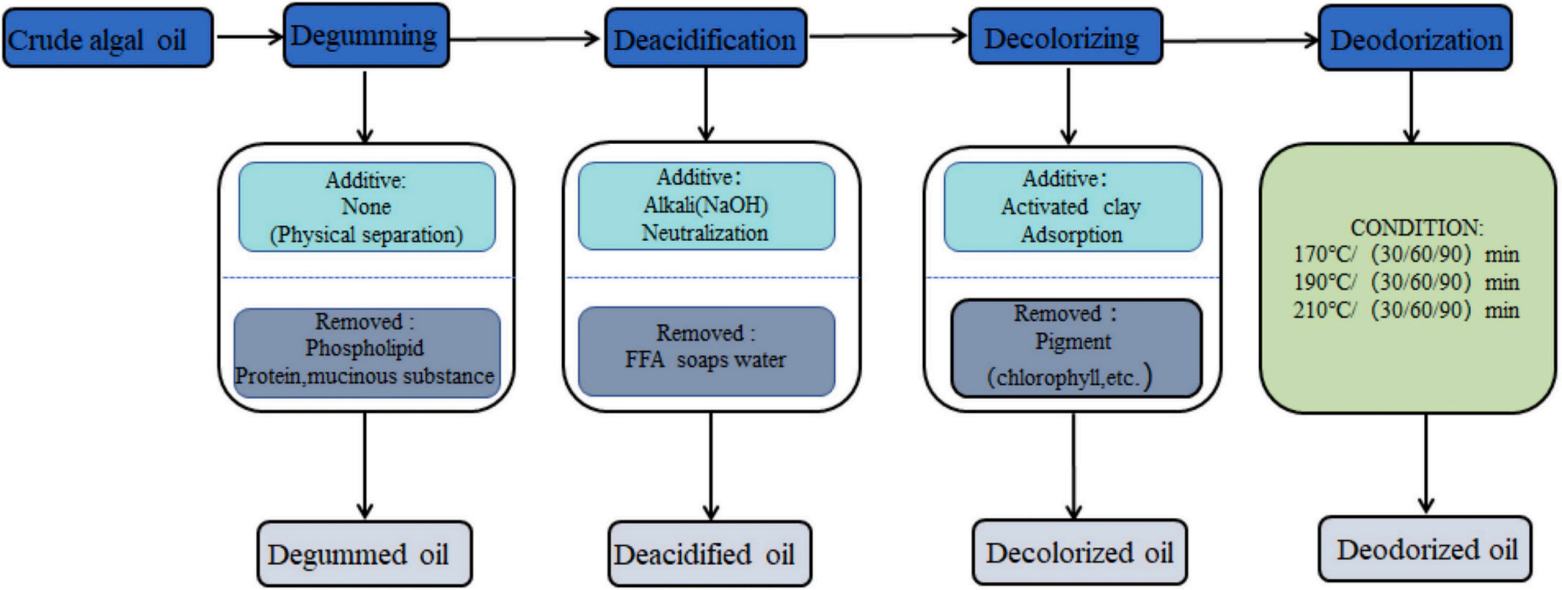
Schematic diagram of the refining process for *Schizochytrium* oil.

Decolorized oil (50 g) was placed in a 500 mL three-necked flask connected to a deodorization apparatus. The system was evacuated to below 1 kPa. Water vapor was continuously introduced at a flow rate of approximately 1.0 mL/min (liquid water equivalent) from the onset of heating to maintain an oxygen-free atmosphere and prevent oxidative degradation. The oil was then heated from room temperature to the target temperatures (190 °C, 210 °C, and 230 °C) at a rate of approximately 15 °C/min, as monitored by a thermocouple. Upon reaching the target temperature, the oil was held for 30, 60, or 90 min while maintaining the same water vapor flow. After deodorization, the oil was cooled to room temperature under vacuum, and the vacuum was then released to obtain the deodorized oil. All deodorized oil samples were stored at 4 °C under nitrogen protection until further analysis. The complete refining process is illustrated in Fig. 1.

### 2.3 Sensory evaluation

Descriptive sensory analysis was conducted based on the method described by Gao et al. (2024),with modifications tailored to the present study. Sensory evaluators were trained according to ISO 8586:2023. The sensory evaluation panel was composed of trained personnel with long-term experience in the evaluation of edible oil flavor. The sensory panel consisted of 5 males and 7 females, aged 20–30. The ethical approval was not required by national laws, and all panelists provided written informed consent. All personal data were anonymized and kept strictly confidential, and participants were informed of their right to withdraw from the evaluation at any time without consequence*.* Oil samples (20 mL) were placed in randomly labeled cups and heated to 40 °C to release the aroma. Panelists rated the intensity of six flavor attributes: fishy flavor, sour flavor, fresh flavor, clarity, color, and off flavor, using a 10-point scale (1 = lowest, 10 = highest). Each sample was evaluated 3 times repeatedly，and the final score for each attribute was obtained by calculating the average of all panelists ‘scores for this attribute.

### 2.4 Electronic nose detection

The performance of the E-nose (cNOSE-18, Shanghai, China) sensor was shown in Table S1. The method for flavor analysis was modified according to Xu et al. (2016). A 1.0 g sample was accurately weighed into a 20 mL headspace vial (KESD Technology Co., Ltd., Shenzhen, China). The sealed vial was then equilibrated in a water bath (model HH-6, Bangxi Instrument Technology Co. Ltd., Shanghai, China) at 25 °C for 30 min, after which an electronic nose (E-nose) probe was inserted to analyze the flavor composition of the *Schizochytrium* oil. The E-nose was operated using clean, dry air as the carrier gas. The E-nose was operated with a sampling time of 120 s, a flow rate of 0.3 L/min, a waiting time of 10 s, and a cleaning time of 120 s, following the procedure of (Xu et al., 2016). The value of sensor signal after stabilization was selected.

### 2.5 HS–SPME/GC–MS analysis of volatile compounds

Volatile components were extracted using headspace solid-phase microextraction coupled with gas chromatography–mass spectrometry (HS-SPME/GC–MS). Following the method of Callejón et al. (2016) with slight modifications, a 5.0 g oil sample was accurately weighed into a 20 mL headspace vial sealed with an aluminum cap. Subsequently, 10 μL of 2,4,6-trimethylpyridine (1.0 mg/mL) was added as an internal standard. The concentration of volatile compounds was calculated using Eq. (2-3):

(2-3) $$C_{i}=\frac{A_{i}}{A_{0}}\times C_{0}$$

In the formula:C_i_ is the concentration of a target volatile compound (mg/kg),A_i_ is its corresponding chromatographic peak area,A_0_ is the peak area of the internal standard (2,4,6-trimethylpyridine),C_0_ is the concentration of the internal standard (mg/kg).

The divinylbenzene/carboxene/polydimethylsiloxane fiber (50 μm/30 μm coating, 1 cm length) (Supelco Inc., Bellefonte, PA, USA) was used to extract the volatile components. After preheating at 60 °C for 30 min, SPME fiber was inserted into the headspace, adsorbed for 15 min at 60 °C, and immediately desorbed at 250 °C for 3 min.

The volatiles were separated on a HP-5MS capillary column (30.0 m × 250 μm × 0.25 μm, Supelco, USA). Helium was used as a carrier gas at a flow rate of 0.8 mL/min. The column temperature was initially held at 4 °C for 1 min, ramped to 150 °C at 8 °C/min, then to 250 °C at 6 °C/min, and held for 6 min. Electron ionization (EI) mass spectra were recorded in full-scan mode at 70 eV across a mass range of 33–500 *m*/*z*. The interface and source temperatures were equaled at 250 °C and 230 °C, respectively.

### 2.6 Relative odor activity value (rOAV)

ROAV was calculated by dividing the concentration of a specific compound by its threshold value and was used to measure the contribution of the compound to the *Schizochytrium* oil flavor (Youfeng et al., 2023). Compounds with an rOAV greater than 1 are considered to contribute to the *Schizochytrium* oil flavor. A larger rOAV indicates that the compound was contributing more to the *Schizochytrium* oil flavor (Zhang et al., 2020). The odor thresholds (Ti) for the volatile compounds were obtained from published literature on edible oil flavor analysis (Czerny et al., 2008). The rOAV for each compound was calculated using Eq. (2-4):

(2-4) $$\mathrm{rOV}A_{i}=\frac{C_{i}}{T_{i}}$$

In the formula:rOA_i_ is the relative odor activity value of compound i,C_i_ is its concentration (mg/kg),T_i_ is the odor threshold of the compound (mg/kg).

### 2.7 Volatile metabolomics analysis

Raw data underwent preprocessing using MS-DIAL 4.70 software, which included peak filtering, peak identification, peak alignment, normalization, and standardization. This yielded a data matrix containing information such as mass-to-charge ratio (*m*/*z*), retention time (RT), and peak intensity for metabolites. The processed data from MS-DIAL 4.70 were then exported, organized, and subjected to PCA and OPLS-DA analysis on the Maiwei Cloud platform. Finally, substances were screened based on the results of multivariate statistical analysis, in combination with FC values, *P*-values, and VIP analysis outcomes.

### 2.8 Statistical analysis

All measurements were performed in triplicate, with results expressed as mean ± standard deviation. Data analysis was conducted using SPSS and Excel 2019 software, including: one-way analysis of variance (ANOVA), Duncan's multiple range test for significant differences (lowercase letters indicate significant differences, *P* < 0.05), correlation analysis, and principal component analysis. Origin 2019 was used for graphing and standard curve linear fitting analysis.

## 3 Results and discussion

### 3.1 Sensory evaluation of *Schizochytrium* oil

**Fig. 2:**
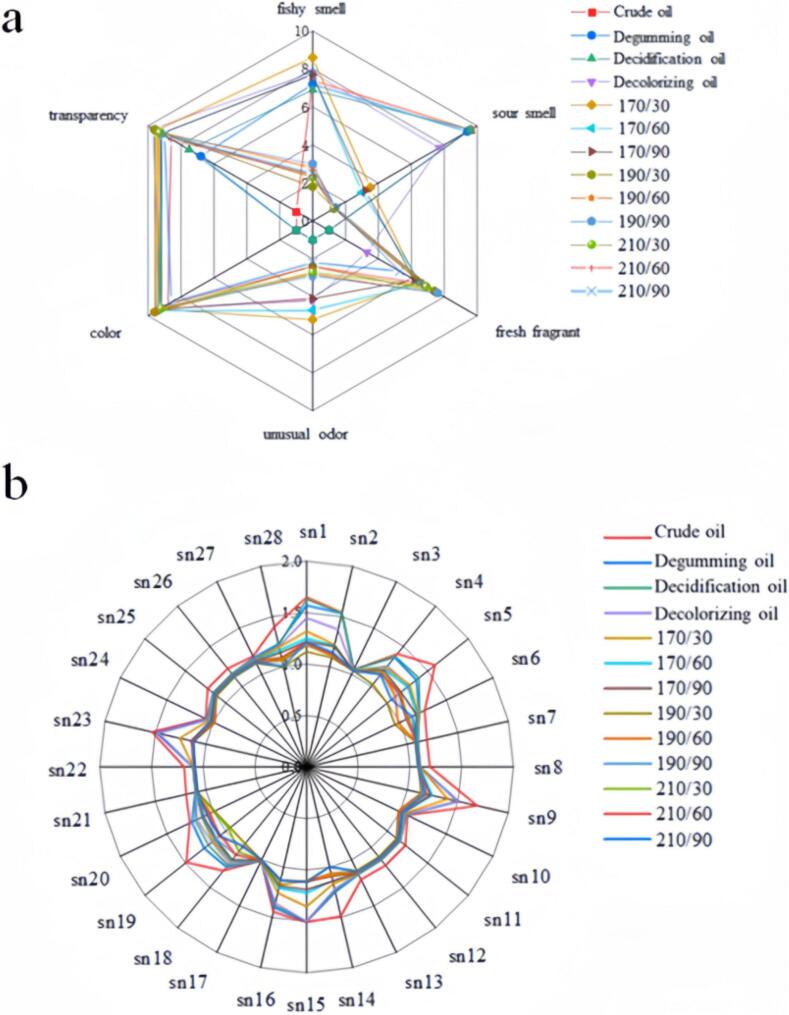
Radar chart of sensory evaluation (a), Electronic nose test results (b).

Fig. 2a shows the sensory properties of algal oil samples during refining, including fishy flavor, sour flavor, fresh flavor, clarity, color, and off flavor. Crude oil scored highest for sourness (9.6) and fishiness (7.4). It appeared deep orange-yellow and turbid. These attributes were linked to short-chain aldehydes (e.g., hexanal) and ketones (e.g., 3-octen-2-one) from lipoxygenase activity and autoxidation. Residual phospholipids and pigments also contributed to its poor clarity. Degumming and deacidification marginally improved sourness and fishiness. However, they significantly enhanced transparency with scores of 6.9 and 7.5. These processes removed phospholipids and free fatty acids. Although these compounds were not major odor contributors, their elimination as oxidative precursors may benefit downstream flavor control. After decolorization, color improved markedly to 9.3. Sourness slightly decreased to 7.7. In contrast, the fishy odor score increased to 7.8. This was likely due to selective adsorption. Bleaching clay preferentially trapped polar macromolecules such as pigments and oxidized polymers. These compounds may have previously masked fishy notes. Meanwhile, the clay exhibited weaker affinity for 3-octen-2-one, a low-molecular-weight and highly volatile fishy odorant (Tiomo et al., 2025). Removal of the masking matrix thus led to a demasking effect, making the fishy odor more pronounced.

Deodorization was the most critical step. Treatment at 170 °C had limited effects. This temperature was insufficient to vaporize key off-flavor compounds such as short-chain aldehydes and ketones. Optimal performance was achieved at 190 °C. After 30 min, fishy and sour odors were largely eliminated. Their scores dropped to 1.8 and 1.3, respectively. A light pleasant aroma emerged with a score of 7.4. This precise thermal input enabled efficient volatilization under vacuum. It also remained below thresholds for triacylglycerol degradation and Maillard reactions (SoYoon et al., 2022). Under these conditions, trace desirable terpenes and lactones became perceptible once dominant off-odors were removed. At 210 °C, the pleasant aroma slightly decreased to 6.8. This suggested that excessive thermal load induced mild lipid autoxidation or Strecker degradation. Trace secondary oxidation products such as 2,4-decadienal were generated. Due to their extremely low odor thresholds, these compounds partially counteracted the pleasant aroma.

### 3.2 Electronic nose detection results of *Schizochytrium* oil

Electronic nose responses at different refining stages are shown in Fig. 2b. In crude oil, sensors sn1, sn2, sn5, sn14, and sn15 exhibited high responses. This indicated the presence of short-chain alkanes and nitrogen-containing compounds. Sensors sn9, sn19, and sn23 also showed elevated responses. This suggested significant contributions from aldehydes, ketones, benzene, and olefins. These findings mirrored the sensory profile of crude oil, where sour and fishy odors were dominant. After degumming, deacidification, and decolorization, responses for nitrogen-containing compounds, short-chain alkanes, olefins, and aromatics generally decreased. This reflected the partial removal of these volatiles. The sensory evaluation revealed an increase in fishy odor after decolorization. However, the electronic nose detected no corresponding reduction in aldehyde- or ketone-sensitive sensors. This discrepancy suggested that selective adsorption of masking compounds contributed to the sensory change, rather than removal of the fishy odorants themselves.

Deodorization at 170 °C caused minor changes in volatile composition. Sensors sn1, sn9, sn15, and sn23 showed negligible response variations. This aligned with the sensory observation that treatment at this temperature had limited effects on sour and fishy odors. At 190 °C, sensor responses across most channels dropped sharply. This indicated effective removal of undesirable volatiles. This sharp reduction coincided with the sensory finding that fishy and sour odors were largely eliminated. A light pleasant aroma emerged at this temperature. At 210 °C, sensors sensitive to short-chain alkanes, alcohols, ketones, and nitrogen-containing compounds showed a slight rebound. This suggested that excessive thermal treatment may have promoted the regeneration or formation of certain odorants. This rebound corresponded with the sensory result that the pleasant aroma slightly decreased at 210 °C.

### 3.3 Qualitative analysis of volatile substances of *Schizochytrium* oil

The qualitative analysis of volatile substances in *Schizochytrium* oil at different refining stages is shown in Table 1. A total of 89 compounds were detected. These included ketones, alcohols, hydrocarbons, aldehydes, esters, terpenes, heterocyclic compounds, phenols, and aromatic hydrocarbons. Compounds with rOAV >1 were considered key flavor contributors. In this study, 28 volatile compounds were identified as key contributors to the flavor profile of *Schizochytrium* oil.

**Table 1:** The primary flavor compounds in *Schizochytrium* oil.

Compounds	Threshold(mg/kg)		Crude oil(mg/kg)	Degreasing oil	Deacidification Oil	Decolorizing oil	170 °C/30 min	170 °C/60 min	170 °C/90 min	190 °C/30 min	190 °C/60 min	190 °C/90 min	210 °C/30 min	210 °C/60 min	210 °C/90 min
(Z)-6-Nonanal	0.00014	Content (mg/kg)	0.17 ± 0.00^a^	0.16 ± 0.00^b^	0.16 ± 0.00^b^	0.16 ± 0.00^b^	–	–	–	–	–	–	–	–	–
		OAV	1214.29	1142.86	1142.86	1142.86	0	0	0	0	0	0	0	0	0
3-Octen-2-one	0.00003	Content (mg/kg)	0.02 ± 0.00^c^	0.02 ± 0.00^c^	0.02 ± 0.00^c^	0.37 ± 0.02^a^	0.20 ± 0.02^b^	0.18 ± 0.01^b^	0.17 ± 0.01^b^	0.01 ± 0.00^c^	0.02 ± 0.00^c^	0.01 ± 0.00^c^	0.01 ± 0.00^c^	0.02 ± 0.00^c^	0.02 ± 0.00^c^
		OAV	666.67	666.67	666.67	12,333.33	6666.67	6000	5666.67	333.33	666.67	333.33	333.33	666.67	666.67
2,2,6-Trimethylcyclohexanone	0.0001	Content (mg/kg)	1036	0.08 ± 0.00^a^	0.08 ± 0.00^a^	0.07 ± 0.00^a^	0.02 ± 0.00^b^	–	–	–	–	–	–	–	–
		OAV	800	800	700	200	0	0	0	0	0	0	0	0	0
trans-2-Octen-1-ol	0.02	Content (mg/kg)	0.23 ± 0.02^a^	0.25 ± 0.01^a^	0.24 ± 0.00^a^	0.24 ± 0.01^a^	0.11 ± 0.00^b^	0.12 ± 0.01^b^	0.09 ± 0.00^bc^	0.04 ± 0.00^d^	0.08 ± 0.00^c^	0.07 ± 0.00^c^	0.06 ± 0.00^c^	0.07 ± 0.00^c^	0.06 ± 0.00^c^
		OAV	11.5	12.5	12	12	5.5	6	4.5	2	4	3.5	3	3.5	3
Benzyl alcohol	0.1	Content (mg/kg)	0.31 ± 0.01^a^	0.32 ± 0.01^a^	0.32 ± 0.00^a^	0.32 ± 0.02^a^	0.15 ± 0.01^b^	0.13 ± 0.00^b^	0.13 ± 0.01^b^	0.03 ± 0.00^c^	0.05 ± 0.00^c^	0.04 ± 0.00^c^	0.03 ± 0.00^c^	0.04 ± 0.00^c^	0.04 ± 0.00^c^
		OAV	3.1	3.2	3.2	3.2	1.5	1.3	1.3	0.3	0.5	0.4	0.3	0.4	0.4
Tridecane	0.042	Content (mg/kg)	0.14 ± 0.00^b^	0.14 ± 0.01^b^	0.14 ± 0.01^b^	0.14 ± 0.01^b^	0.15 ± 0.00^a^	0.13 ± 0.00^c^	0.13 ± 0.00^c^	0.12 ± 0.00^d^	0.13 ± 0.01^c^	0.13 ± 0.01^c^	0.12 ± 0.00^d^	0.13 ± 0.00^c^	0.13 ± 0.00^c^
		OAV	3.33	3.33	3.33	3.33	3.57	3.1	3.1	2.86	3.1	3.1	2.86	3.1	3.1
3-Hexenal	0.16	Content (mg/kg)	810	4.48 ± 0.13^a^	4.52 ± 0.07^a^	4.51 ± 0.05^a^	2.01 ± 0.15^b^	0.69 ± 0.04^c^	0.58 ± 0.02^d^	0.68 ± 0.02^c^	0.04 ± 0.00^e^	0.07 ± 0.00^e^	0.04 ± 0.00^e^	0.06 ± 0.00^e^	0.08 ± 0.00^e^
		OAV	28	28.25	28.19	12.56	4.31	3.63	4.25	0.25	0.44	0.25	0.38	0.5	0.25
(Z)-3-Hexenal	0.004	Content (mg/kg)	0.20 ± 0.01^a^	0.21 ± 0.02^a^	0.21 ± 0.02^a^	0.20 ± 0.01^a^	0.03 ± 0.00^b^	0.04 ± 0.00^b^	0.04 ± 0.00^b^	–	0.05 ± 0.00^b^	–	–	–	–
		OAV	50	52.5	52.5	50	7.5	10	10	0	12.5	0	0	0	0
trans-4-Decenal	0.025	Content (mg/kg)	0.02 ± 0.01^b^	0.02 ± 0.01^b^	0.02 ± 0.00^b^	0.02 ± 0.00^b^	0.04 ± 0.00^a^	–	–	–	–	–	–	–	–
		OAV	0.8	0.8	0.8	0.8	1.6	0	0	0	0	0	0	0	0
Benzaldehyde	0.35	Content (mg/kg)	0.54 ± 0.02^a^	0.53 ± 0.03^a^	0.54 ± 0.03^a^	0.47 ± 0.02^b^	0.32 ± 0.01^c^	0.29 ± 0.01^cd^	0.28 ± 0.02^d^	0.11 ± 0.01^ef^	0.15 ± 0.01^e^	0.16 ± 0.00^e^	0.13 ± 0.00^e^	0.14 ± 0.01^e^	0.14 ± 0.01^e^
		OAV	1.54	1.51	1.54	1.34	0.91	0.83	0.8	0.31	0.43	0.46	0.37	0.4	0.4
(E,E)-2,4-Decadienal	0.07	Content (mg/kg)	–	–	–	–	0.09 ± 0.00^a^	0.09 ± 0.00^a^	0.09 ± 0.00^a^	–	–	0.05 ± 0.00^b^	0.04 ± 0.00^b^	0.09 ± 0.00^a^	–
		OAV	0	0	0	0	1.29	1.29	1.29	0	0	0.71	0.57	1.29	0
γ-hexalactone	0.26	Content (mg/kg)	0.20 ± 0.00^cd^	0.21 ± 0.00^cd^	0.21 ± 0.00^cd^	0.17 ± 0.00^d^	0.21 ± 0.00^cd^	0.20 ± 0.00^cd^	0.23 ± 0.00^c^	0.31 ± 0.00	0.32 ± 0.00^a^	0.32 ± 0.00^a^	0.27 ± 0.00^b^	0.29 ± 0.00^b^	0.34 ± 0.00^a^
		OAV	0.77	0.81	0.81	0.65	0.81	0.77	0.88	1.15	1.23	1.23	1.04	1.12	1.31
Cyclohexanecarboxylic acid ethyl ester	0.0016	Content (mg/kg)	0.25 ± 0.01^a^	0.25 ± 0.01^a^	0.24 ± 0.02^b^	0.24 ± 0.00^b^	0.06 ± 0.00^c^	0.04 ± 0.00^c^	0.06 ± 0.00^c^	–	0.01 ± 0.00^d^	–	–	–	–
		OAV	156.25	156.25	150	150	37.5	25	37.5	0	6.25	0	0	0	0
2-Methylbutyl isobutyrate	0.014	Content (mg/kg)	0.13 ± 0.01^b^	0.13 ± 0.01^b^	0.13 ± 0.00^b^	0.13 ± 0.00^b^	–	–	–	–	0.02 ± 0.00^a^	–	–	–	–
		OAV	9.29	9.29	9.29	9.29	0	0	0	0	1.43	0	0	0	0
Beta-caryophyllene	0.034	Content (mg/kg)	0.53 ± 0.03^a^	0.51 ± 0.03^a^	0.52 ± 0.01^a^	0.2 ± 0.01^b^	0.1 ± 0.00^c^	0.08 ± 0.00^c^	0.05 ± 0.00^d^	0.08 ± 0.00^c^	0.04 ± 0.00^d^	0.05 ± 0.00^d^	0.07 ± 0.00^c^	0.05 ± 0.00^d^	0.06 ± 0.00^cd^
		OAV	15.59	15	15.29	5.88	2.94	2.35	1.47	2.35	1.18	1.47	2.06	1.47	1.76
Z)-3,7-Dimethyl-1,3,6-octadekatrien	0.034	Content (mg/kg)	0.53 ± 0.01^a^	0.49 ± 0.02^a^	0.51 ± 0.01^a^	0.51 ± 0.01^a^	0.33 ± 0.01^b^	0.31 ± 0.02^b^	0.29 ± 0.1^b^0	0.22 ± 0.00^c^	0.11 ± 0.00^e^	0.07 ± 0.00^f^	0.15 ± 0.00^d^	0.13 ± 0.00^d^	0.14 ± 0.00^d^
		OAV	15.59	14.41	15	15	9.71	9.12	8.53	6.47	3.24	2.06	4.41	3.82	4.12
L-borneol	0.048	Content (mg/kg)	0.30 ± 0.02^a^	0.30 ± 0.00^a^	0.30 ± 0.01^a^	–	–	–	–	–	–	–	–	–	–
		OAV	6.25	6.25	6.25	0	0	0	0	0	0	0	0	0	0
Linalool	0.006	Content (mg/kg)	0.04 ± 0.00^b^	0.04 ± 0.00^b^	0.03 ± 0.00^c^	0.03 ± 0.00^c^	0.04 ± 0.00^b^	0.04 ± 0.0^b^0	0.05 ± 0.00^a^	0.04 ± 0.00^b^	0.04 ± 0.00^b^	0.04 ± 0.00^b^	0.03 ± 0.00^c^	0.03 ± 0.00^c^	0.02 ± 0.00^d^
		OAV	6.67	6.67	5	5	6.67	6.67	8.33	6.67	6.67	6.67	5	5	3.33
5-Methyl-2-(1-methylethyl)-cyclohexanone	0.0047	Content (mg/kg)	0.13 ± 0.01^a^	0.14 ± 0.01^a^	0.14 ± 0.01^a^	0.13 ± 0.00^a^	0.13 ± 0.01^a^	0.12 ± 0.00^a^	0.10 ± 0.00^b^	0.03 ± 0.00^c^	–	–	–	–	–
		OAV	27.66	29.79	29.79	27.66	27.66	25.53	21.28	6.38	0	0	0	0	0
Borneol	0.18	Content (mg/kg)	0.30 ± 0.03^a^	0.31 ± 0.02^a^	0.30 ± 0.03^a^	0.30 ± 0.01^a^	0.12 ± 0.01^b^	0.10 ± 0.00^b^	0.13 ± 0.00^b^	–	–	0.01 ± 0.00^c^	–	–	
		OAV	1.67	1.72	1.67	1.67	0.67	0.56	0.72	0	0	0.06	0	0	0
5-Ethyl-2-(5H)-furanone	0.0097	Content (mg/kg)	2.70 ± 0.05^a^	2.63 ± 0.04^a^	2.65 ± 0.06^a^	1.38 ± 0.05^b^	0.89 ± 0.02^c^	0.77 ± 0.04^d^	0.87 ± 0.05^c^	0.03 ± 0.00^ef^	0.06 ± 0.00^e^	0.06 ± 0.00^e^	0.08 ± 0.00^e^	0.08 ± 0.00^e^	0.07 ± 0.00^e^
		OAV	278.35	271.13	273.2	142.27	91.75	79.38	89.69	3.09	6.19	6.19	8.25	8.25	7.22
2-Ethoxy-3-methylpyrazine	0.0008	Content (mg/kg)	0.06 ± 0.00^c^	0.06 ± 0.00^c^	0.06 ± 0.00^c^	0.06 ± 0.00^c^	0.07 ± 0.00^b^	0.09 ± 0.00^a^	0.07 ± 0.00^b^	–	–	–	–	–	–
		OAV	75	75	75	75	87.5	112.5	87.5	0	0	0	0	0	0
Dihydro-2-methyl-3(2H)-furanone	/	Content (mg/kg)	0.22 ± 0.01^a^	0.21 ± 0.02^a^	0.22 ± 0.00^a^	0.11 ± 0.01^b^	0.01 ± 0.00^c^	–	–	–	–	–	–	–	
		OAV	8.2	7.83	8.2	4.1	0.37	0	0	0	0	0	0	0	0
Prenyl mercaptan	/	Content (mg/kg)	0.07 ± 0.00^b^	0.07 ± 0.00^b^	0.08 ± 0.00^a^	0.07 ± 0.00^b^	0.04 ± 0.00^c^	0.04 ± 0.00^c^	–	–	–	–	–	–	
		OAV	1.4	1.4	1.6	1.4	0.8	0.8	0	0	0	0	0	0	0
Guaiacol	0.0016	Content (mg/kg)	0.02 ± 0.00^b^	0.03 ± 0.00^a^	–	–	–	–	–	–	–	–	–	–	
		OAV	12.5	18.75	0	0	0	0	0	0	0	0	0	0	0
Phenol	0.03	Content (mg/kg)	0.49 ± 0.01^a^	0.49 ± 0.01^a^	0.49 ± 0.02^a^	0.48 ± 0.02^a^	0.38 ± 0.02^b^	0.34 ± 0.03^b^	0.33 ± 0.01^b^	0.11 ± 0.00^e^	0.17 ± 0.01^d^	0.16 ± 0.01^d^	0.20 ± 0.02^c^	0.22 ± 0.01^c^	0.22 ± 0.01^c^
		OAV	16.33	16.33	16.33	16	12.67	11.33	11	3.67	5.67	5.33	6.67	7.33	7
Hexanal	4.5	Content (mg/kg)	0.28 ± 0.01^a^	0.25 ± 0.02^a^	0.27 ± 0.02^a^	0.26 ± 0.01^a^	0.19 ± 0.01^b^	0.20 ± 0.02^b^	0.19 ± 0.00^b^	0.02 ± 0.00^cd^	0.03 ± 0.00^cd^	0.05 ± 0.00^c^	0.06 ± 0.00^c^	0.06 ± 0.00^c^	0.06 ± 0.00^c^
		OAV	0.06	0.06	0.06	0.06	0.04	0.04	0.04	0	0.01	0.01	0.01	0.01	0.01
Aminaldehyde	1	Content (μg/kg)	0.45 ± 0.02^b^	0.46 ± 0.01^b^	0.44 ± 0.01^bc^	0.41 ± 0.00^c^	0.53 ± 0.02^a^	0.50 ± 0.01^a^	0.47 ± 0.01^b^	0.07 ± 0.00^e^	0.09 ± 0.00^de^	0.10 ± 0.00^de^	0.13 ± 0.01^d^	0.13 ± 0.00^d^	0.12 ± 0.01^d^
		OAV	0.45	0.46	0.44	0.41	0.53	0.5	0.47	0.07	0.09	0.1	0.13	0.13	0.12

Note: “/” indicates that the detection limit is not reached.

In crude oil, 3-octen-2-one, with an rOAV of 666.67, and several short-chain aldehydes, such as (Z)-6-nonenal with an rOAV of 1214.29, exhibited high rOAV values. These compounds were identified as the primary sources of the sour and fishy odors detected in sensory evaluation. Degumming and deacidification did not substantially change the composition or rOAV of these key flavor compounds. This finding aligned with the sensory observation that sour and fishy odors showed limited improvement after these steps. Decolorization removed six key compounds, including guaiacol and l-borneol, through adsorption by bleaching clay. This removal accounted for the reduction in sour and rancid sensory notes, with sourness decreasing from 9.6 to 7.7. A notable finding was the dramatic increase in the rOAV of 3-octen-2-one after decolorization, which surged to 12,333.33, making it the dominant odorant. Given its extremely low odor threshold of 3.0 × 10^−5^ mg/kg, even trace amounts could produce a significant sensory impact. This molecular change explained the intensified fishy odor detected by the sensory panel, with fishiness increasing from 7.4 to 7.8. The electronic nose data corroborated this finding, as aldehyde- and ketone-sensitive sensors (sn9, sn19, sn23) showed no corresponding reduction in response after decolorization.

These three lines of evidence supported a selective adsorption mechanism. Hussin et al. (2011) documented that bleaching clay preferentially removed masking compounds such as pigments and oxidized polymers. In contrast, it exhibited a relatively weaker affinity for low molecular weight, highly volatile compounds like 3-octen-2-one.This selective adsorption likely contributed to a perceptual demasking effect. In this study, although the volatile species directly adsorbed onto the clay were not the primary analytical targets, the convergent evidence derived from sensory evaluation, electronic nose, and rOAV analysis strongly supported the existence of this demasking phenomenon. Specifically, the sensory panel recorded an intensified fishy note after decolorization. Meanwhile, the electronic nose showed no corresponding reduction in aldehyde/ketone sensors, and the rOAV of 3-octen-2-one surged dramatically. These observations were all consistent with the selective retention of this key odorant. This observation aligned with the matrix effect, which demonstrated that non-volatile matrix components could significantly modulate the release and perception of flavor-active compounds (Xiao et al., 2023). Taken together, these findings suggested that the demasking effect might play a non-negligible role in the sensory changes observed during decolorization, and future targeted add-back experiments with extracted adsorbates would provide further validation of this effect.

Deodorization at 170 °C moderately reduced the number and rOAV of key flavor compounds. However, 22 contributors remained, with 3-octen-2-one still predominant at an rOAV of 6666.67. This limited reduction was consistent with sensory evaluation, where sour and fishy odors were only slightly diminished. The electronic nose also showed negligible response variations in sensors sn1, sn9, sn15, and sn23. The flavor profile underwent a significant transformation after deodorization at 190 °C. The rOAV of 3-octen-2-one plummeted to 333.33. Other compounds with sweet and fresh aromas then defined the sensory profile. These included terpenes such as β-caryophyllene with an rOAV of 2.35 and lactones such as γ-hexalactone with an rOAV of 1.15. This compositional shift correlated perfectly with sensory evaluation. Fishy and sour odors were largely eliminated, with scores dropping to 1.8 and 1.3, respectively. A light pleasant aroma emerged with a score of 7.4. The electronic nose also recorded a sharp drop in sensor responses across most channels, confirming the effective removal of undesirable volatiles.

### 3.4 Volatile metabolomics analysis of *Schizochytrium* oil

To understand how deodorization affects the volatile composition of *Schizochytrium* oil, a non-targeted volatile metabolomics approach was employed. Crude oil (MY) and oil samples deodorized at 190 °C/30 min, 190 °C/90 min, 210 °C/30 min, and 210 °C/90 min were analyzed. This strategy aimed to identify key differential metabolites and to elucidate the evolution mechanisms of off-lavor compounds through multi-dimensional data correlation.

#### 3.4.1 Analytical stability and data quality

**Fig. 3:**
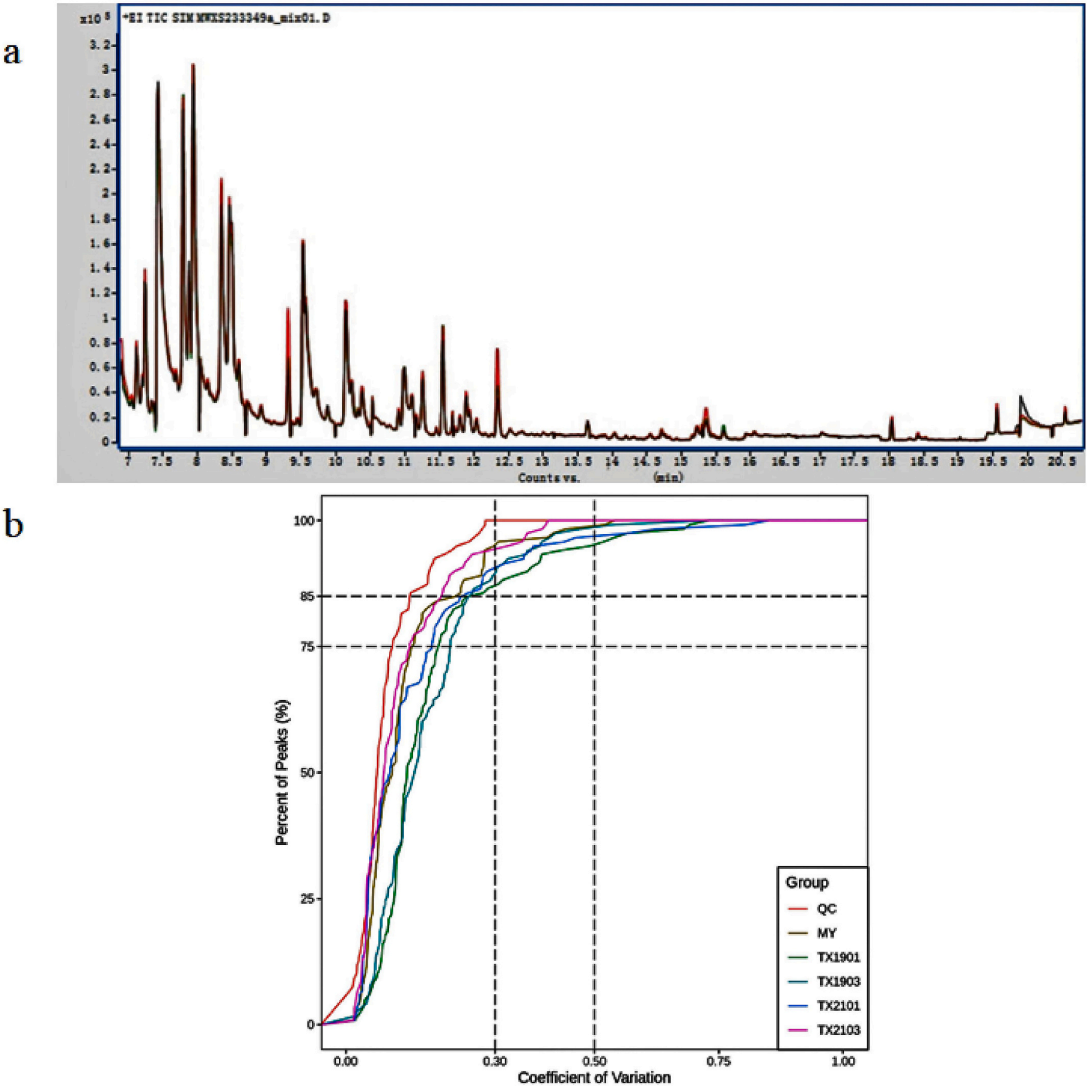
Overlay of total ion flow for mass spectrometry detection of QC samples (a),Distribution of sample CV (b).

Quality control (QC) samples were used to monitor the reliability of non-targeted metabolomics data. The total ion chromatograms of QC samples showed high consistency in retention times and response intensities (Fig. 3a). This indicated excellent instrumental stability.

A coefficient of variation (CV) analysis was performed for all detected metabolites. Over 75% of the metabolites had CV values below 0.3 (Fig. 3b). This met the stringent criteria for non-targeted metabolomics analysis. These results provided a high-quality data foundation for subsequent multivariate statistical analysis.

#### 3.4.2 Multivariate statistical analysis of metabolic profiles

**Fig. 4:**
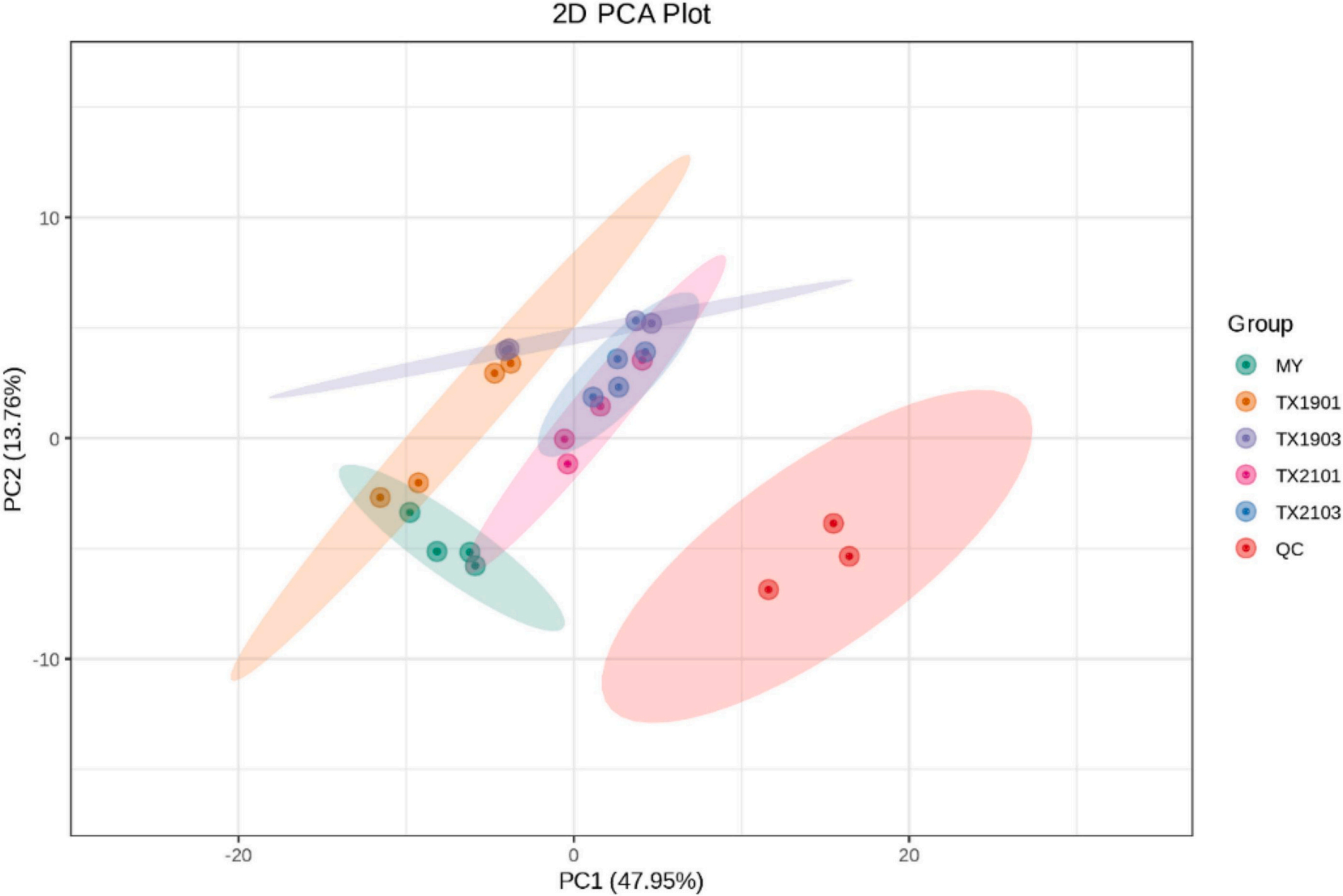
Plot of PCA scores for each group of algal oil samples.

Principal Component Analysis (PCA) was first applied to visualize overall metabolic differences among samples (Abdi & Williams, 2010). The PCA score plot (Fig. 4) showed that the first principal component (PC1) explained 47.95% of the variance. PC1 clearly separated crude oil from all deodorized oil samples. This indicated that deodorization was the primary driver of global changes in the volatile metabolic profile. The second principal component (PC2) explained 13.76% of the variance. PC2 further distinguished samples treated at 190 °C from those treated at 210 °C. This suggested that processing intensity led to more subtle variations in the metabolic profile.

**Fig. 5:**
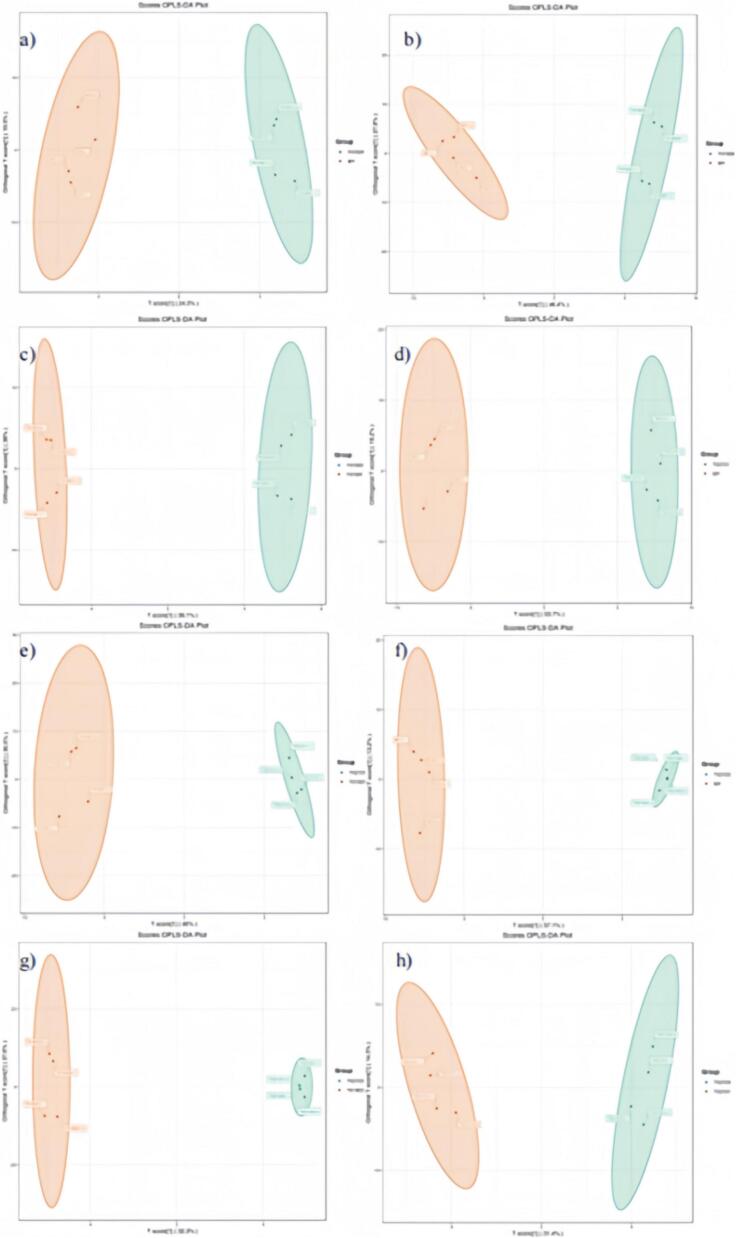
OPLS-DA model for algal oil samples between groups Note: Figure a) shows crude oil VS 190 °C/30 min, b) shows crude oil VS 190 °C/90 min, c) shows 190 °C/30 min VS 190 °C/90 min, d) shows crude oil VS 210 °C/30 min e) shows 210 °C/30 min vs. 190 °C/30 min, f) shows crude oil vs. 210 °C/90 min g) shows 210 °C/90 min vs. 190 °C/90 min, h) shows 210 °C/30 min vs. 210 °C/90 min.

Orthogonal Partial Least Squares-Discriminant Analysis (OPLS-DA) was then employed to maximize inter-group separation and screen for key differential metabolites (Trygg & Wold, 2002). All OPLS-DA models for key group comparisons showed clear inter-group separation (Fig. 5). Model parameters (R^2^Y and Q^2^) all exceeded 0.84 (Table S2). This indicated good model fit and predictive ability.

To further validate the OPLS-DA models, K-fold cross-validation (7 folds) with 200 permutation tests was performed for each pairwise comparison (Groups A–H). As shown in Fig. S1, all permuted Q^2^ values (blue bars) were consistently below zero, while the original Q^2^ values (>0.84) were significantly higher, confirming that the models were statistically significant and not overfitted (N et al., 2015). Given that permutation tests are more robust for small-sample datasets and do not rely on theoretical distribution assumptions, we consider the permutation test results to be the more reliable validation metric for our current dataset.

#### 3.4.3 Screening of differential metabolites

Based on the Variable Importance in Projection (VIP > 1.0) from the OPLS-DA model, combined with the *p*-value (< 0.05) from univariate analysis and fold change (FC > 2 or < 0.5), the significantly differential metabolites between groups were screened and identified (Table 2). These differential metabolites constitute the molecular basis for the flavor changes occurring during the deodorization process. Among them, eight volatile metabolites—namely 3-octen-2-one, trans-2-octen-1-ol, benzyl alcohol, 5-ethyl-2(5H)-furanone, beta-caryophyllene, phenol, 2-ethylpiperidine, and 4-methylthiazole—were identified as potential biomarkers for monitoring deodorization efficiency.

**Table 2:** Volatile differential metabolites in groups of algal oil samples.

Group and code	Matter	Classification	Formula	CAS	VIP	P-value	FC
*Group A*							
XMW0226	4,6-Dimethyl-2-heptanone	ketone	C_9_H_18_O	19,549-80-5	1.37	0.12	8.12
KMW0230	3-Octen-2-one	ketone	C_8_H_14_O	1669-44-9	2.00	0.00	0.49
KMW0294	trans-2-Octen-1-ol	Pure	C_8_H_16_O	18,409-17-1	1.81	0.01	0.18
KMW0323	Benzyl alcohol	Pure	C_7_H_8_O	100-51-6	1.79	0.01	0.10
XMW0072	α,α-Dimethyl-4-methylene-cyclohexanemethanol	Pure	C_10_H_18_O	7299-42-5	2.02	0.00	0.51
XMW0196	4-Methyl-decane	hydrocarbons	C_11_H_24_	2847-72-5	1.81	0.00	0.22
XMW0237*041	5-Ethyl-5-methyl-decane	hydrocarbons	C_13_H_28_	17,312-74-2	2.01	0.00	3.55
XMW0649	2,6-Dimethylnonane	hydrocarbons	C_11_H_24_	17,302-28-2	1.29	0.02	6.85
NMW0735	1-Hydroxy-2-propane	hydrocarbons	C_3_H_6_O_2_	116-09-6	1.31	0.01	0.01
IS-15	(R)-(−)2H4-Apigenin	Terpenes	C_10_H_10_D_4_O	IR-37376	2.02	0.00	0.06
WMW0215*018	Beta-caryophyllene	Terpenes	C_10_H_16_	13,877-91-3	1.37	0.00	0.15
KMW0235*018	(Z)-3,7-Dimethyl-1,3,6-octadekatrien	Terpenes	C_10_H_16_	3338-55-4	1.32	0.00	0.42
KMW0334	5-Methyl-2-(1-methylethyl)-cyclohexanone	Terpenes	C_10_H_18_O	10,458-14-7	1.29	0.01	0.23
KMW0539	5-Ethyl-2-(5H)-furanone	Heterocyclic	C_6_H_8_O_2_	2407-43-4	1.48	0.02	0.01
XMW0267	2-Ethylpiperidine	Heterocyclic	C_7_H_15_N	1484-80-6	1.32	0.02	0.42
XMW0430	2-Piperidineimine	Heterocyclic	C_5_H_10_N_2_	22,780-54-7	1.31	0.12	0.45
KMW0175	Phenol	phenol	C_6_H_6_O	108-95-2	1.74	0.01	0.23

*Group B*							
XMW0226	4,6-Dimethyl-2-heptanone	ketone	C_9_H_18_O	19,549-80-5	1.42	0.01	13.20
KMW0230	3-Octen-2-one	ketone	C_8_H_14_O	1669-44-9	1.46	0.01	0.50
KMW0294	trans-2-Octen-1-ol	Pure	C_8_H_16_O	18,409-17-1	1.38	0.01	0.31
KMW0323	Benzyl alcohol	Pure	C_7_H_8_O	100-51-6	1.87	0.00	0.13
XMW0072	α,α-Dimethyl-4-methylene-cyclohexanemethanol	Pure	C_10_H_18_O	7299-42-5	1.45	0.00	0.49
XMW0237*041	5-Ethyl-5-methyl-decane	hydrocarbons	C_13_H_28_	17,312-74-2	1.46	0.00	5.58
XMW0196	4-Methyl-decane	hydrocarbons	C_11_H_24_	2847-72-5	1.36	0.02	0.20
XMW0281	5-Methyl-decane	hydrocarbons	C_11_H_24_	13,151-35-4	1.43	0.00	0.44
XMW0649	2,6-Dimethylnonane	hydrocarbons	C_11_H_24_	17,302-28-2	1.46	0.00	7.11
NMW0735	1-Hydroxy-2-propane	hydrocarbons	C_3_H_6_O_2_	116-09-6	1.44	0.00	0.01
WMW0215*018	Beta-caryophyllene	Terpenes	C_10_H_16_	13,877-91-3	1.28	0.02	0.10
KMW0235*018	(Z)-3,7-Dimethyl-1,3,6-octadekatrien	Terpenes	C_10_H_16_	3338-55-4	1.34	0.01	0.13
XMW1444*107	3-Isopropyl-6-methyl-2-cyclohexen-1-one	Terpenes	C_10_H_16_O	499-74-1	1.36	0.06	0.47
KMW0389*092	Borneol	Terpenes	C_10_H_18_O	507-70-0	1.49	0.00	0.03
XMW0267	2-Ethylpiperidine	Heterocyclic	C_7_H_15_N	1484-80-6	1.43	0.00	0.31
Group and Code	Matter	Classification	Formula	CAS	VIP	P-value	FC
KMW0175	Phenol	phenol	C_6_H_6_O	108-95-2	1.62	0.01	0.33

*Group C*							
KMW0235*018	(Z)-3,7-Dimethyl-1,3,6-octadekatrien	Terpenes	C_10_H_16_	3338-55-4	1.46	0.03	0.32
KMW0539	5-Ethyl-2-(5H)-furanone	Heterocyclic	C_6_H_8_O_2_	2407-43-4	1.12	0.05	2.20

*Group D*							
XMW0226	4,6-Dimethyl-2-heptanone	ketone	C_9_H_18_O	19,549-80-5	1.07	0.11	12.18
KMW0230	3-Octen-2-one	ketone	C_8_H_14_O	1669-44-9	1.04	0.08	0.49
KMW0294	trans-2-Octen-1-ol	Pure	C_8_H_16_O	18,409-17-1	1.30	0.01	0.26
KMW0323	Benzyl alcohol	Pure	C_7_H_8_O	100-51-6	1.23	0.03	0.10
XMW0072	α,α-Dimethyl-4-methylene-cyclohexanemethanol	Pure	C_10_H_18_O	7299-42-5	1.35	0.01	3.05
XMW0196	4-Methyl-decane	hydrocarbons	C_11_H_24_	2847-72-5	1.87	0.00	0.17
XMW0237*041	5-Ethyl-5-methyl-decane	hydrocarbons	C_13_H_28_	17,312-74-2	1.35	0.00	5.08
XMW0281	5-Methyl-decane	hydrocarbons	C_11_H_24_	13,151-35-4	1.34	0.00	0.44
XMW0649	2,6-Dimethylnonane	hydrocarbons	C_11_H_24_	17,302-28-2	1.36	0.00	7.62
NMW0735	1-Hydroxy-2-propane	hydrocarbons	C_3_H_6_O_2_	116-09-6	1.24	0.00	0.01
KMW0158	Benzaldehyde	aldehyde	C_7_H_6_O	100-52-7	1.08	0.05	0.24
WMW0215*018	Beta-caryophyllene	Terpenes	C_10_H_16_	13,877-91-3	1.16	0.01	0.13
KMW0235*018	(Z)-3,7-Dimethyl-1,3,6-octadekatrien	Terpenes	C_10_H_16_	3338-55-4	1.21	0.00	0.29
NMW0034	1,3,3-Trimethyl-bicyclo[2.2.1]heptan-2-one	Terpenes	C_10_H_16_O	1195-79-5	1.36	0.03	2.03
KMW0539	5-Ethyl-2-(5H)-furanone	Heterocyclic	C_6_H_8_O_2_	2407-43-4	1.42	0.00	0.03
XMW0267	2-Ethylpiperidine	Heterocyclic	C_7_H_15_N	1484-80-6	1.05	0.00	0.36
KMW0499	4-Methylthiazole	Heterocyclic	C_4_H_5_NS	693-95-8	2.05	0.03	0.01
KMW0175	Phenol	phenol	C_6_H_6_O	108-95-2	1.21	0.01	0.41

*Group E*							
XMW0072	α,α-Dimethyl-4-methylene-cyclohexanemethanol	Pure	C_10_H_18_O	7299-42-5	1.14	0.04	5.95
KMW0306	Undecane	hydrocarbons	C_11_H_24_	1120-21-4	1.40	0.01	3.33

*Group F*							
XMW0226	4,6-Dimethyl-2-heptanone	ketone	C_9_H_18_O	19,549-80-5	1.43	0.02	17.26
KMW0294	trans-2-Octen-1-ol	Pure	C_8_H_16_O	18,409-17-1	1.25	0.01	0.26
KMW0323	Benzyl alcohol	Pure	C_7_H_8_O	100-51-6	1.31	0.00	0.13
XMW0072	α,α-Dimethyl-4-methylene-cyclohexanemethanol	Pure	C_10_H_18_O	7299-42-5	1.35	0.04	2.54
XMW0237*041	5-Ethyl-5-methyl-decane	hydrocarbons	C_13_H_28_	17,312-74-2	1.02	0.00	6.60
XMW0281	5-Methyl-decane	hydrocarbons	C_11_H_24_	13,151-35-4	1.18	0.00	0.44
XMW0649	2,6-Dimethylnonane	hydrocarbons	C_11_H_24_	17,302-28-2	1.09	0.01	8.12
KMW0235*018	(Z)-3,7-Dimethyl-1,3,6-octadekatrien	Terpenes	C_10_H_16_	3338-55-4	1.36	0.02	1.35
Group and Code	Matter	Classification	Formula	CAS	VIP	P-value	FC
NMW0034	1,3,3-Trimethyl-bicyclo[2.2.1]heptan-2-one	Terpenes	C_10_H_16_O	1195-79-5	1.38	0.01	3.05
KMW0539	5-Ethyl-2-(5H)-furanone	Heterocyclic	C_6_H_8_O_2_	2407-43-4	1.26	0.00	0.03

The key off-flavor compounds identified by rOAV (Table 1) were significantly downregulated after deodorization, with FC values typically below 0.1. For example, fishy and metallic 3-octen-2-one showed FC = 0.49 in Group A (crude oil vs. 190 °C/30 min), consistent with the sensory reduction of fishy odor from 7.4 to 1.8. Likewise, caramel-like 5-ethyl-2(5H)-furanone decreased with FC = 0.01, corresponding to the elimination of sour odor from 9.6 to 1.3. The electronic nose further confirmed this removal, as aldehyde- and ketone-sensitive sensors (sn9, sn19, sn23) showed sharply reduced responses at 190 °C. Notably, several additional substances were significantly removed after deodorization but were not flagged as key contributors in the targeted rOAV analysis (Table 1). These included solvent-like benzyl alcohol with an FC of 0.10 in Group A, earthy and musty 2-ethylpiperidine with an FC of 0.42, and the terpene β-caryophyllene with an FC of 0.15. Their individual rOAV values were below 1. Nevertheless, their removal contributed to the overall sensory improvement. This finding highlights a key advantage of untargeted metabolomics. It captures compositional changes that collectively shape the flavor matrix and influence perceptual masking or synergy among odorants.

Comparing samples deodorized at 190 °C and 210 °C (Table 2, Groups E and G), the relative abundance of 3-octen-2-one was higher at 210 °C than at 190 °C. The FC was 0.49 in Group A, whereas no significant downregulation was observed in Group E. This temperature-induced rebound explained the sensory observation. The pleasant aroma decreased from 7.4 at 190 °C to 6.8 at 210 °C. The electronic nose data in Section 3.2 detected a corresponding rebound in aldehyde- and ketone-sensitive sensors at 210 °C, confirming that excessive thermal treatment promoted the regeneration of these off-odorants.

At both 190 °C and 210 °C, extending deodorization from 30 min to 90 min caused only minor additional changes in the volatile profile. See Table 2, Groups C and H. For example, (Z)-3,7-dimethyl-1,3,6-octadekatrien showed an FC of 0.32 in Group C, and 5-ethyl-2(5H)-furanone showed an FC of 2.20. These small shifts indicate that prolonged heating marginally altered specific terpenes and furanones but did not substantially improve or degrade the overall flavor quality. This aligns with the sensory evaluation in Section 3.1. The scores for fishy and sour odors at 190 °C/90 min were 1.7 and 1.2, respectively, nearly identical to those at 190 °C/30 min which were 1.8 and 1.3.

#### 3.4.4 Hierarchical cluster analysis

**Fig. 6:**
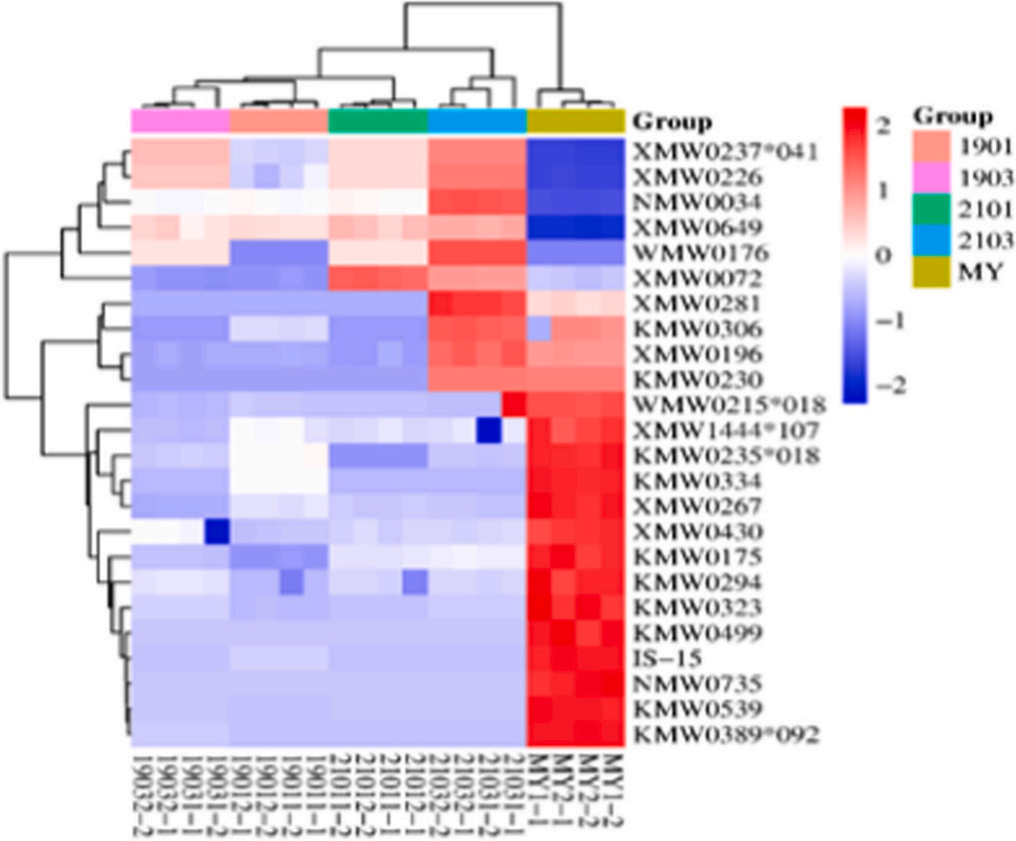
Clustered heat map of the relative amounts of major differential compounds in all.

To clearly visualize the variation trends of differential metabolites across sample groups, hierarchical cluster analysis was performed using the MetaboAnalyst platform. The results are shown in Fig. 6. After deodorization, the volatile metabolite profile of *Schizochytrium* oil changed markedly. The clustered heatmap was divided into two main categories. The blue regions indicated metabolites that were downregulated in deodorized oils compared to crude oil. The red regions indicated metabolites that were upregulated.

The heatmap showed that compounds such as 4 methylthiazole, toluene, benzaldehyde, and trans 2 octen 1 ol were generally downregulated after deodorization. This pattern directly matched the sensory elimination of fishy and sour odors. It also matched the sharp drop in electronic nose sensor responses to aldehydes, ketones, and nitrogen containing compounds. Notably, 3-octen-2-one and 5-ethyl-2(5H)-furanone had the highest rOAV values in crude oil according to Table 1. They were among the most strongly downregulated metabolites in the heatmap. Their near-complete removal at 190 °C/30 min explained why the sensory panel scored fishiness as low as 1.8 at this condition. Some alkanes, heterocyclic compounds, and terpenes were upregulated after deodorization. β-Caryophyllene and γ-hexalactone had low odor thresholds and showed increased abundance at 190 °C, contributing woody/spicy and sweet/coconut notes respectively. This upregulation results from two concurrent effects: a demasking effect that improves perceptual access to residual pleasant compounds, and thermally induced formation of certain volatiles, e.g., γ-Hexalactone increased from 0.20 to 0.31 μg/kg at 190 °C/30 min, a change consistent with lactonization of hydroxy fatty acid precursors (e.g., 4-hydroxydecanoic acid) under thermal conditions. This mechanism was well-documented in dairy and lipid systems, where hydroxy fatty acids cyclized to form γ-lactones upon mild heating (Alewijn et al., 2006). Notably, the deodorization conditions employed in this study were non-oxidative in nature, which thermodynamically favored intramolecular cyclization over oxidative degradation pathways and thus indirectly supported the plausibility of this mechanism(Bassem et al., 2022). Given that the present study focused primarily on profiling volatile metabolites via HS-SPME/GC-MS, the specific pathways underlying the formation of these upregulated compounds, including the possible lactonization of hydroxy fatty acid precursors, merited further investigation in subsequent studies. The net abundance thus reflects a balance between heat-driven generation and evaporative removal (Bassem et al., 2022).

Integrating volatilomics and flavoromics revealed a biphasic mechanism governing flavor evolution during *Schizochytrium* oil deodorization. At moderate temperatures from 170 °C to 190 °C, vacuum assisted thermal energy vaporized low molecular weight off-odorants. These included unsaturated ketones and short chain aldehydes. Volatilomics confirmed their removal. This created a perceptual space in which residual terpenes and lactones became detectable. This demasking effect explains why a pleasant aroma emerged at 190 °C but not at lower temperatures. At 210 °C, a distinct phenomenon occurred. Thermal energy induced secondary lipid oxidation or Strecker degradation. This led to regeneration of certain aldehydes and ketones. Their absolute concentrations remained low. However, their extremely low odor thresholds enabled sensory detection. This thermal rebound mechanism explains why higher temperature did not improve flavor but instead reduced sensory quality. The transition between the two regimes occurred near 190 °C. Below this threshold, volatile removal dominated. Above it, heat induced formation offset removal. Thus, the integration of volatilomics and flavoromics defines the optimal deodorization condition as the point where stripping efficiency is maximized before thermally driven regeneration becomes active.

## 4 Conclusion

By integrating sensory evaluation with untargeted volatile metabolomics, this study reveals for the first time a biphasic thermal transition mechanism governing flavor evolution during *Schizochytrium* oil deodorization: below 190 °C, volatile off-odorants are efficiently stripped, whereas above 210 °C, thermal degradation triggers a rebound of aldehydes and ketones that compromises flavor quality. This defined temperature boundary provides a theoretical foundation for precise control of deodorization processes. Resolving this biphasic mechanism further uncovered two critical phenomena that traditional methods fail to capture. First, selective adsorption by bleaching clay during decolorization creates a “demasking” effect, paradoxically intensifying fishy odor. Second, several non-key odorants with rOAV < 1 (e.g., 2-ethylpiperidine, β-caryophyllene) are substantially removed under optimal deodorization, demonstrating that matrix synergy significantly contributes to overall flavor perception. These findings are inaccessible to targeted rOAV analysis alone, highlighting the irreplaceable advantage of integrating untargeted metabolomics with sensory flavoromics. Based on these mechanistic and phenomenological insights, we propose a practical biomarker panel of eight volatile metabolites for monitoring deodorization endpoints, and identify 190 °C/30 min as the optimal condition. This multidimensional framework not only provides a scientific basis for precise flavor regulation in *Schizochytrium* oil refining, but is also transferable to process optimization of other high-value edible oils. Direct confirmation of the lactonization pathway through targeted precursor analysis is warranted in future studies.

## Authors contribution

Mingjie Li, Guanzhong Xiang, Hanle Yuan, Xiaolong Yang and Yunpeng Ding performed the experiment, obtained, analyzed, and compiled data, and Mingjie Li wrote the manuscript. Pao Gao and Xinghe Zhang carried out the idea, Jiaojiao Yin and Lei Tan designed this study, Zhuolong Guan and Hanhua Lou supervised this study, Dongping He provided funds, and Wu Zhong was responsible for the planning, design, and implementation of the entire study and revised the manuscript. All authors have read and agreed to the published version of the manuscript.

## CRediT authorship contribution statement

**Mingjie Li:** Writing – original draft. **Guanzhong Xiang:** Data curation. **Hanle Yuan:** Formal analysis. **Yuqi Zhang:** Methodology. **Yunpeng Ding:** Investigation. **Xiaolong Yang:** Methodology. **Pan Gao:** Supervision. **Jiaojiao Yin:** Software. **Xinghe Zhang:** Project administration. **Zhuolong Guan:** Supervision. **Lei Tan:** Resources. **Hanhua Lou:** Resources. **DongPing He:** Funding acquisition. **Wu Zhong:** Conceptualization. **Yacheng Hao:** Visualization.

## Funding

Wuhan Polytechnic University introduction scientific research start-up project (2024RZ020); This work was supported by the Hubei Provincial Key Research and Development Program (2023BBB134).

## Declaration of competing interest

The authors declare that they have no known competing financial interests or personal relationships that could have appeared to influence the work reported in this paper.

## Data Availability

The data presented in this study are available on request from the corresponding author.

## Appendix A Supplementary data

Supplementary material 1: Supplementary Table S1 (electronic nose sensors), Table S2 (OPLS-DA model parameters), and Figure S1 (permutation test results) for algal oil samples between groups.
