# Supplementary material for: Decoding flavor evolution during refining of DHA-rich Schizochytrium oil: a multidimensional framework integrating sensory evaluation and volatile metabolomics
Source: Food Chem X. 2026 Jul 28;38:104255. doi: 10.1016/j.fochx.2026.104255 (PMC13450594; doi:10.1016/j.fochx.2026.104255)
Supplement: Supplementary file 1 [file mmc1.docx]

Supplementary Materials

Table S1 Electronic nose sensors and their response substances

| Sensor | **Target Analytes** | Sensor | **Target Analytes** |
| --- | --- | --- | --- |
| S1 | **Short-Chain Alkane** | S15 | Sensitive to **nitrogen-containing compounds** |
| S2 | **Carbon-Containing Compounds, etc** | S16 | Sensitive to **sulfur compounds** |
| S3 | **Hydrogen** | S17 | **Hydrogen-containing substances** |
| S4 | **Sulfur Compounds** | S18 | **Alcohols, certain organic solvents, etc.** |
| S5 | **Nitrogen-Containing Compounds** | S19 | Sensitive to **alcohols, aldehydes, ketones, and aromatic compounds** |
| S6 | **Aldehydes and Ketones** | S20 | Sensitive to **short-chain alkanes** |
| S7 | **Flammable Short-Chain Alkanes** | S21 | Sensitive to **short-chain alkanes** |
| S8 | **Liquefied Petroleum Gas** | S22 | VOC |
| S9 | **Alkanes, Alcohols, Ketones, etc** | S23 | Sensitive to **alkanes, alkenes, aromatic hydrocarbons, etc** |
| S10 | **Hydrogen and Hydrogen-Containing Substances** | S24 | Sensitive to **alkanes, alkenes, aromatic hydrocarbons, etc** |
| S11 | **Alkanes, Carbon Monoxide, etc** | S25 | Sensitive to **alkanes, carbon monoxide, alkenals, alcohols, nitrogen oxides, ketones, aldehydes, etc** |
| S12 | **Certain Organic Solvents** | S26 | Sensitive to **certain organic solvents** |
| S13 | **Short-Chain Alkane Species** | S27 | **Sulfides, nitrides, carbides, hydrocarbons, and nitrogen oxides, etc** |
| S14 | **Short-Chain Alkanes** | S28 | **Short-chain alkanes** |

Table S2 Evaluation parameters of OPLS-DA model for algal oil samples between groups

| Number | Group | R^2^X | R^2^Y | Q^2^ |
| --- | --- | --- | --- | --- |
| A | Crude oil and190℃/30 min | 0.439 | 0.991 | 0.916 |
| B | Crude oil and 190℃/90 min | 0.742 | 0.989 | 0.957 |
| C | 190℃/30 min and190℃/90 min | 0.767 | 0.998 | 0.845 |
| D | Crude oil and 210℃/30 min | 0.719 | 0.996 | 0.975 |
| E | 210℃/30 min and190℃/30 min | 0.766 | 0.995 | 0.968 |
| F | Crude oil and 210℃/90 min | 0.702 | 0.999 | 0.987 |
| G | 210℃/30 min and190℃/90 min | 0.871 | 0.999 | 0.848 |
| H | 210℃/30 min and210℃/90 min | 0.458 | 0.992 | 0.854 |

## Figure S1 Permutation test results for OPLS-DA models of algal oil samples between groups

Note: Figure a) shows crude oil VS 190°C/30 min, b) shows crude oil VS 190°C/90 min,

c) shows 190°C/30 min VS 190°C/90 min, d) shows crude oil VS 210°C/30 min

e) shows 210℃/30 min vs. 190 ℃/30 min, f) shows crude oil vs. 210℃/90 min

g) shows 210℃/90 min vs. 190 ℃/90 min, h) shows 210℃/30 min vs. 210℃/90 min.
